# Impact of Direct Transport to Thrombectomy-Capable Center vs. Nearby/Distant Local Stroke Centers on Stroke Outcome in Patients Undergoing Thrombectomy: A Real-Life Study

**DOI:** 10.3390/jpm14040395

**Published:** 2024-04-08

**Authors:** Cristina del Toro-Pérez, Laura Amaya-Pascasio, Antonio Arjona-Padillo, Patricia Martínez-Sánchez

**Affiliations:** 1 Stroke Centre, Department of Neurology, Torrecárdenas University Hospital, 04009 Almería, Spain; 2 Faculty of Health Sciences, CEINSA (Center of Health Research), University of Almería, 04120 Almería, Spain

**Keywords:** ischemic stroke, acute stroke, mechanical thrombectomy, transfer model, direct transfer, secondary transfer

## Abstract

Our aim was to compare the stroke outcomes of a direct transfer (DT) to a thrombectomy-capable center vs. initial care at two local stroke centers: a nearby hospital (NH, 36 km) and a distant hospital (DH, 113 km). Patients who underwent a mechanical thrombectomy were analyzed (February 2017–October 2021), and the outcome was considered favorable if the modified Rankin scale (mRS) score was ≤ 2 at three months. A total of 300 patients were included, 55 of which were transferred from the NH and 58 from the DH. There was a difference in the median (IQR) transfer time of 39 min between the hospitals (149 min for the NH vs. 188 min for the DH, *p* = 0.003). After adjusting for confounding variables, a secondary transfer from the DH, compared to a DT, was associated with a lower functional independence: mRS score ≤ 2 (OR = 0.37, 95% CI = 0.14–0.97, *p* = 0.043), without significant differences in the mortality between the groups. These differences were not observed in patients from the NH. Conclusions: A secondary transfer from a distant hospital was associated with a poorer functional outcome at 3 months. This unfavorable outcome was not observed among patients transferred from a nearby hospital. These findings highlight the importance of categorizing the suitability of one transfer model over another based on the proximity of hospitals to the thrombectomy center, but also in accordance with organizational and geographic characteristics that vary within each health region.

## 1. Introduction

The treatment of an acute ischemic stroke with a large vessel occlusion (LVO) currently consists of intravenous thrombolysis (IVT) and mechanical thrombectomy (MT) [1]. MT is at present available only in specialized thrombectomy-capable centers (TCs) [1]. Different organizational models are used for patients suspected of an acute stroke. While some patients are transferred directly to a TC to reduce the time to MT [2], others are transported to the nearest hospital for an initial assessment and possible earlier intravenous fibrinolysis (IVT) before being transferred to the TC in the case of a LVO [3].

At present, according to the current scientific evidence, there is no established model for transferring patients who have experienced an acute stroke that is superior to others [4,5]. The effectiveness of transfer models varies depending on the unique characteristics of each healthcare area, including the geographical factors, available resources, and interhospital organization [4,5]. As a result, a global consensus on the best transfer model has not been achieved [4,5]. Indeed, a recent large clinical trial showed that direct transportation to a TC, compared to the closest local stroke center (LSC), did not reduce the chance of disability at 90 days (RACECAT clinical trial) [6]. It has been proposed that a TC distance greater than 20 km or a transfer time of more than 15 min may determine the desirability of a direct transfer over a secondary transfer [7].

We hypothesized that a secondary transfer to a TC may affect the outcome of patients with a LVO and that the impact varies according to the distance traveled. Our aim was to compare the impact on the stroke outcome of a direct transfer (DT) to the TC vs. initial care in one of two LSCs with different distances to the TC under the same resources and organizational system: a nearby hospital (NH) (36 km) and a distant hospital (DH) (113 km).

## 2. Methods

### 2.1. Patient Population

**Figure 1:**
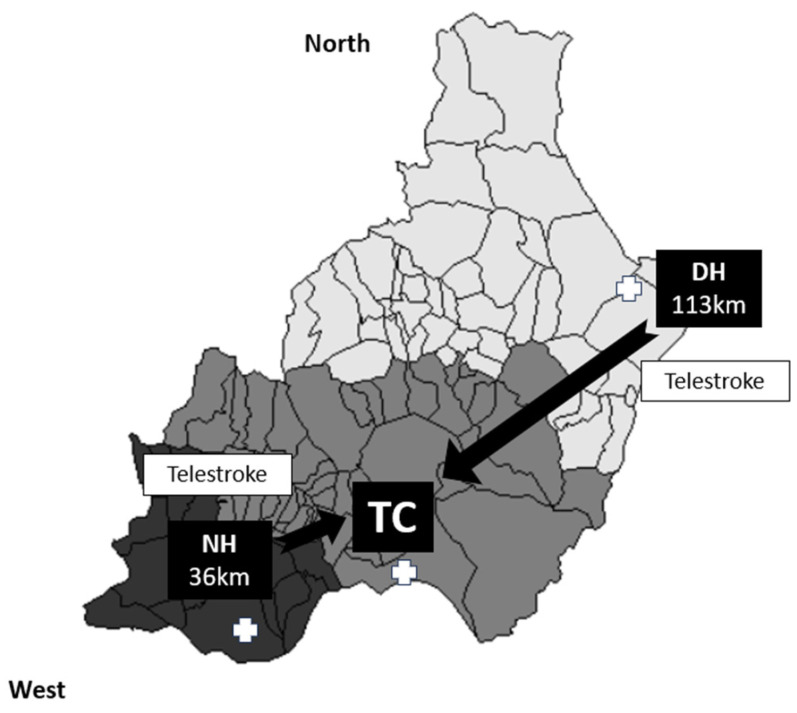
Organization of the health area in the province of Almería. Patients with a suspected acute ischemic stroke are transferred to the nearest hospital (light grey zone for the DH, medium grey zone for the TC, and dark grey zone for the DH), with a subsequent transfer to the TC if an LVO is detected for MT. Both the NH and DH have a 24/7 telestroke service.

We conducted a retrospective cross-sectional study of patients aged ≥18 years who had experienced an anterior- or posterior-circulation acute ischemic stroke and underwent MT with or without previous IVT, in accordance with the current international guidelines [1]. This study was conducted in Almería between February 2017 and October 2021. Almería (Andalusia) is a province in southern Spain that serves a total population of 739,293 inhabitants. There are three stroke centers: a TC and two LSCs (both telestroke centers), with one located 36 km away from the TC (the NH) and another situated 113 km away (the DH) (Figure 1). Stroke patients from the TC health area are referred using a DT model, while patients from the other two health areas within the province (approximately 60% of the population) are transferred using a secondary transfer model.

The variables considered in this study encompassed demographic data, vascular risk factors and comorbidities, previous treatments, blood biomarkers, stroke characteristics, and the etiology according to the TOAST classification [2]. Additionally, the National Institutes of Health Stroke Scale (NIHSS) score on admission, the reperfusion treatments (MT and IVT), the time periods (onset-to-door, onset-to-groin, and door-to-groin times), recanalization by modified TICI grades, the number of passes during the MT, and the 3-month outcome measured by the modified Rankin scale (mRS) score were also included in the analysis. The presence of in-hospital complications was retrieved, including the occurrence of a hemorrhagic transformation during the first 36 h (parenchymal hematoma type 1—PH1—or type 2—PH2) according to the ECASS classification [3], brain edema, a craniectomy, renal failure, lower respiratory tract infections, and urinary tract infections.

### 2.2. Outcome Parameters

The primary outcome was a favorable functional outcome at 3 months, defined as an mRS score of 0–2. The secondary outcome was death due to any cause at 3 months, defined as an mRS score of 6.

### 2.3. Data Analysis

The statistical analysis was conducted using IBM SPSS Statistics software, version 25.0 (IBM Corp., Armonk, NY, USA). Continuous variables were presented as the mean (standard deviation [SD]) or median (interquartile range [IQR]) and compared using Student’s *t*-test or the Mann–Whitney test, as appropriate, or ANOVA and the Kruskal–Wallis test for multiple comparations. Group comparisons were analyzed using the chi-squared test or Fisher’s exact test for dichotomous variables. The relationship between the transferring model (a variable with three categories according to the first hospital: TC, NH, and DH) and the 3-month outcome was assessed using two multivariate binary logistic regression models. Baseline and clinical variables with a *p*-value < 0.2 in the bivariate analysis, with the mRS score as the dependent variable, were considered potential confounders, and were included in the multivariate analysis. Additionally, variables that exhibited significant differences in the bivariate analysis between transfer model groups were also included. A backward procedure was followed as the modeling strategy, using the log likelihood ratio test to evaluate the goodness of fit and compare nested models. Variables that, when eliminated, resulted in a change of ≥15% in the odds ratios (ORs) were considered confounding variables. The ORs and their corresponding 95% confidence intervals (CIs) were used to assess the strength of association. A *p*-value of <0.05 was considered statistically significant.

### 2.4. Ethical Issues

This study protocol adhered to the ethical guidelines of the 1975 Declaration of Helsinki and all subsequent amendments. The project received approval from the clinical research ethics committee of the Torrecárdenas University Hospital. The data collected for the study were processed in compliance with the General Data Protection Regulation (EU) 2016/679 of the European Parliament and of the Council of 27 April 2016.

## 3. Results

A total of 300 patients were included. A failure to follow-up with some patients resulted in the loss of data regarding the 3-month outcome; therefore, these data were available for a total of 279 patients. The baseline and stroke-related characteristics of the included patients are listed in Table 1. The median (IQR) age was 72 (19) and 59.7% of the patients were men. Fifty-five patients were transferred from the NH and 58 from the DH following a secondary transfer model. The median (IQR) secondary transfer time from the LSC to the TC was 169 (98) minutes, with a difference of 39 min between the two groups (149 min for the NH group vs. 188 min for the DH group, *p* = 0.003). The baseline characteristics between the groups based on the transfer method were similar, except for the percentage of patients with *de novo* atrial fibrillation (26.7% for the DT group, 14.5% for the NH transfer group, and 35.4% for the DH transfer group, *p* = 0.031).

There were no statistically significant differences among the groups in the number of patients treated with IVT or the median onset-to-needle time. However, a higher median door-to-needle time was observed for the DH group (60 min for the DH group compared to 43 min for the DT and NH groups, *p* < 0.001). A lower median door-to-groin time was found for the DH group (64 min for the DH group vs. 95 min for the DT and NH groups, *p* < 0.001).

A higher percentage of symptomatic hemorrhagic transformations was observed in patients transferred from the NH and DH (23.6% for the NH group vs. 22.4% for the DH group vs. 12.3% for the DT group, *p* = 0.004).

The main procedural variables, complications, hospitalization outcomes, and clinical outcomes are summarized in Table 2.

**Figure 2:**
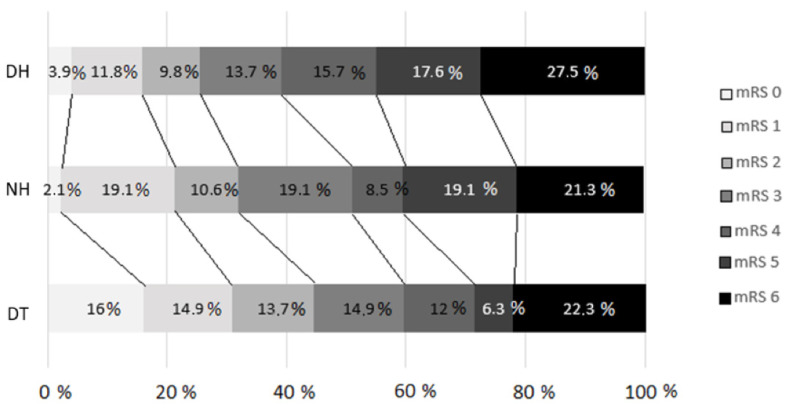
Modified Rankin scale distribution at 90 days (*p* = 0.04) for comparison between groups. DT, direct transfer; NH, nearby hospital; DH, distant hospital. A failure to follow-up with some patients resulted in the loss of data regarding the 3-month prognosis based on the mRS, which was analyzed for a total of 279 patients.

Figure 2 shows the 3-month outcomes assessed by the mRS in the different arms according to the transfer model (*p* = 0.04).

Regarding the primary outcomes, a higher percentage of patients achieved functional independence (mRS score ≤ 2) in the DT group (41.7% for the DT group vs. 27.3% for the NH group vs. 22.4% for the DH group, *p* = 0.027). The multivariate statistical analysis revealed that a secondary transfer from the DH was associated with a lower percentage of patients achieving functional independence (OR = 0.37, 95% CI = 0.14–0.97, *p* = 0.043), with similar mortality rates (mRS score = 6) among the groups. The results of the multivariate analyses for an mRS score ≤ 2 are presented in Table 3.

## 4. Discussion

This real-world study represents a novel endeavor in comparing functional outcomes based on the transfer pattern to a TC, stratified by the distance from the LSCs to the TC under identical resources, protocols, and organizational models. The study enrolled patients who had experienced an acute ischemic stroke and an LVO, underwent MT, and were either directly transferred to the TC or indirectly transferred from LSCs. One LSC was nearby, while the other was distant. A poorer functional prognosis at 3 months was noted in patients who underwent a secondary transfer, but this association was only evident in those transferred from the distant LSC. This unfavorable outcome was driven by a lower percentage of patients achieving functional independence (mRS score = 0–2), while the 3-month mortality rates remained consistent across all the groups. These findings are in line with prior research indicating a better prognosis with a DT to the TCcompared to a secondary transfer, especially from more distant hospitals [4].

The literature lacks consensus regarding whether a DT or what is classically referred to as mothership, or secondary transfer following a drip-and-ship model, is a more optimal transfer model for moving patients to a TC. Some meta-analyses have also observed a more favorable functional prognosis at 3 months in the DT group, whether or not the analysis adjusted for transfer times [5,6,7]. However, these meta-analyses mainly comprised heterogeneous observational studies and did not consider the distance from the LSC. Similar rates of successful recanalization and mortality have been reported between the two evaluated transfer models, which aligns with the findings of our study. In contrast to our findings, with a higher rate of hemorrhagic transformations in patients who were secondarily transferred from any of the regional hospitals, no significant differences in terms of symptomatic intracranial hemorrhages were found in these meta-analyses [5,6,7].

Romoli et al. found that there was a shorter time from stroke onset to reperfusion treatment following a DT, potentially explaining the better prognosis observed in this group [5]. This finding is supported by previous data indicating that, for every hour of delay until recanalization, the likelihood of achieving functional independence decreases by 10–38% [5,6]. In our study, longer door-to-needle, onset-to-groin, and door-to-groin times were also observed in the secondary transfer group, but this was only evident in the subgroup of patients who were transferred from the DH. The onset-to-needle time was similar between the groups, with neither model causing a delay in IVT. It is worth noting that our province implemented a telestroke network via video call in July 2019, providing distant specialized care to patients with a suspected ischemic stroke that were primarily assessed by regional hospitals [8]. Prior to this, the province had a telestroke system based on telephone calls to the on-call referring neurologist. This may have contributed to the similarity in the samples concerning IVT treatments, despite the persistence of longer door-to-needle times in the secondary transfer group. Previous studies have shown longer times to IVT treatment associated with the use of a telestroke service versus an on-site specialist, despite favoring an increase in reperfusion treatment in cases where the patient’s presence is not possible [9].

Two large-scale observational studies conducted in the USA and in Portugal that involved over 1000 patients who experienced an acute stroke also demonstrated better clinical outcomes for the DT model [10,11], as well as another conducted in Germany with a similar sample of patients to our study [12]. Hypothetical bypass modeling for the transferred patients indicated that IVT would be delayed by 12 min, but the MT would be performed 91 min earlier if the patients were directly routed to the TC [11]. A sub-study of the SWIFT PRIME clinical trial showed that patients treated with IVT at an outside hospital had less favorable outcomes than those who received both IVT and an endovascular intervention at the TC. However, the relative benefit from the MT did not differ significantly in the two groups [13]. Other real-life studies with a design similar to ours have not reported significant differences in the functional outcome, substantial recanalization, or symptomatic hemorrhagic transformation between the two transfer models, although the process times were longer in the secondary transfer groups in most cases [14,15,16,17,18,19,20,21].

In some of these observational studies, the secondary transfer model showed a lower percentage of patients receiving IVT [14], while others observed higher rates of IVT in this model [16]. In our cohort, we did not find significant differences in this regard. As expected, real-life studies, including our own, have demonstrated a lower onset-to-groin time in the direct transfer group [14,19]. In a Dutch study, a comparative analysis was conducted by changing the protocol from a secondary to a DT model. Over four years, there were more IVT and MT procedures performed, and both the onset-to-needle and onset-to-groin times decreased, but no specific assessment of the prognosis was reported [19]. None of these studies stratified the data according to the distance from the primary center or the transfer time to the TC in order to assess the specific impact of distance on the transfer models. In some cases, the primary centers were located within less than 17 km [14], while in others, the distance was significantly greater and heterogeneous between centers or not reported [15,16,22]. In a study by Rinaldo et al., the mortality was significantly lower among directly admitted patients than among transferred patients, and an increased distance between the transferring hospital and the TC was associated with an increased risk of mortality [23].

The RACECAT trial, a randomized clinical trial conducted in Catalonia, Spain, was recently published, and it showed similar prognoses and mortality rates between the secondary and DT groups. In this study, the type of transfer was randomized without using prior large-vessel prediction scales, which could have helped in selecting patients with a higher probability of an LVO who are, therefore, more likely to benefit from a DT [24,25,26,27]. Additionally, patients within a 30 km radius of the TC were not included in the study; they were directly referred to the TC, potentially underestimating the benefits of the DT model. This is particularly relevant in other areas such as ours, where patients are usually transferred to primary centers without MT capabilities, despite being within a 30 km radius of the TC, highlighting the importance of stratifying transfer model studies based on the distance from the TC to the LSC. Regarding the availability of a telestroke service in the LSCs in this trial, there was a certain heterogeneity that may have influenced the indication of reperfusion treatments and the time to IVT onset. A subsequent neuroimaging sub-study of the data revealed that patients in the secondary transfer group who underwent vascular imaging at the primary hospital had a significantly higher rate of receiving MT and a shorter door-to-groin time [28]. Similarly, in our study, a shorter median door-to-groin time was observed in the DH group compared to the DT group, possibly because repeat neuroimaging was not required, which led to a shorter time to MT once the TC was reached. A novel aspect of our study is the stratification of prognostic data and variables of interest based on the location of two LSCs in an area with similar resources, protocols, and organizational models. This decision was driven by the recognition of sufficiently heterogeneous geographical characteristics, secondary transport resources, and logistical factors, which may justify the different feasibility of transfer models within the same health area. This hypothesis was confirmed, as statistically significant data indicating a worse functional prognosis for the secondary transfer model were only observed in patients coming from the DH. A possible reason for the varied outcomes between the NH and DH patients might be the difference in the transfer time to the TC. The transfer time differed by nearly 40 min between the two LSCs, which could explain the prognostic disparities, leading to a delay in performing MT for patients from the DH. Moreover, a lower median door-to-needle time and a shorter median onset-to-groin time were observed in the DT and NH groups compared to the DH group, which are both well-known variables associated with the prognosis after an acute stroke [29,30]. In a HERMES collaboration meta-analysis, the onset-to-reperfusion times were significantly shorter in the DT group compared to the secondary transfer group. The rates of functional independence at 3 months declined with a delay in this onset–to-reperfusion time [29]. Future treatments, such as new ways of administering IVT and new treatments that may influence the benefit of MT may change the appropriateness of one organizational model over another, as the need for transport is highly dependent on the treatments available for an ischemic stroke with an LVO and their application [31,32].

These results are of interest, and they challenge previous expert recommendations suggesting that the secondary transfer model might be more suitable for hospital areas located farthest from the TC [3]. Our findings suggest the opposite, possibly due to the significant delay in performing MT caused by the added time of transfer from the DH and the coordination required with the local transfer services in the DH area. This study underscores the importance for each healthcare region to assess its resources comprehensively and in a segmented manner. It highlights the need to plan the most suitable model in collaboration with each primary center and to implement potential improvements tailored to the unique characteristics of each area, recommendations that are likely applicable and relevant to healthcare regions worldwide. Although the scales available for the detection of an LVO do not show a sufficiently high positive predictive ability at the present time to determine, with good fidelity, whether a direct or secondary transfer should be used, they may be of some use in individualizing the transfer of certain patients, especially in the case of hospitals farther away from the TC [1].

This study has some limitations, such as its retrospective nature and the limitation of patient inclusion to those who experienced an acute ischemic stroke with an LVO and underwent MT. Additionally, the sample size was small, which could have influenced the non-difference between the DT group and the secondary-transfer-from-the-NH group. The fact that the patients included in the DT group exclusively belonged to the healthcare region of the TC prevented us from comparing both models in patients from different healthcare regions, limiting the generalizability of our findings. However, it should be noted that several regions belonging to the TC region, which therefore allow for the direct transfer of patients, were at a similar distance away from this center as the nearby and distant hospital areas that followed a secondary transfer model, bringing the comparability of the results in the different health areas closer. Further research with larger sample sizes and multi-center collaborations could provide more robust evidence on the optimal transfer models for acute stroke patients.

## 5. Conclusions

A secondary transfer from a distant hospital was associated with a poorer functional outcome at 3 months in our cohort. This unfavorable outcome was not observed among patients transferred from a nearby hospital. These findings highlight the importance of categorizing the suitability of one transfer model over another based on the proximity to the thrombectomy center, but also in accordance with organizational and geographic characteristics that vary within each health region.

## Figures and Tables

**Table 1:** Baseline and stroke characteristics.

Variable	Total (N = 300)	DT (N = 187)	NH (N = 55)	DH (N = 58)	*p*
**Demographic data and comorbidities**					
Female, *n* (%)	121 (40.3)	77 (41.2)	25 (45.5)	19 (32.8)	0.29
Age, median (IQR), years	72 (19)	72 (20)	72 (20)	70.5 (15)	0.67
Previous mRS score **≤ 1, *n* (%)**	258 (86)	158 (84.5)	50 (90.9)	50 (86.2)	0.07
Smoker, *n* (%)	62 (20.7)	40 (21.4)	11 (20)	11 (18.9)	0.95
Alcoholism, *n* (%)	34 (11.3)	21 (11.3)	6 (10.9)	7 (12.1)	0.90
Drug abuse, *n* (%)	5 (1.6)	4 (2.1)	0 (0)	1 (1.5)	0.56
**Glycemia, median (IQR), mg/dL**	127 (52.5)	127 (61)	127 (61)	126 (50)	0.81
Hypertension, *n* (%)	188 (62.6)	118 (63.1)	32 (58.2)	38 (65.5)	0.91
Dyslipidemia, *n* (%)	130 (43.4)	78 (41.7)	21 (38.2)	31 (53.4)	0.25
Diabetes mellitus, *n* (%)	86 (28.7)	56 (29.9)	15 (27.3)	15 (25.9)	0.84
Ischemic cardiopathy, *n* (%)	45 (15)	32 (17.1)	4 (7.3)	9 (15.5)	0.24
Previous stroke, *n* (%)	27 (9)	17 (9.1)	5 (9.1)	5 (8.6)	0.8
Previous atrial fibrillation, *n* (%)	67 (22.3)	43 (23)	13 (23.6)	11 (19)	0.73
***De novo*** atrial fibrillation *, *n* (%)	81 (27)	50 (26.7)	8 (14.5)	23 (35.4)	0.03
**Prior treatments**					
Statins, *n* (%)	133 (44.3)	77 (41.2)	25 (45.5)	31 (53.4)	0.11
Antiplatelet agents, *n* (%)	103 (34.3)	69 (36.9)	17 (30.9)	17 (29.3)	0.75
Anticoagulants, *n* (%)	39 (13)	25 (13.4)	7 (12.7)	7 (12.1)	0.98
Underdosed vitamin K antagonist **, *n* (%)	18 (6.3)	10 (5.3)	4 (25)	4 (26.7)	0.87
**Vascular territory**					
Anterior circulation, *n* (%)	264 (87.3)	165 (88.2)	48 (87.3)	51 (87.9)	0.88
Left side, *n* (%)	145 (48.7)	88 (47.1)	25 (45.5)	32 (55.2)	0.48
**NIHSS, median (IQR)**	17 (9)	17 (9)	17 (9)	17 (8)	0.67
**ASPECTS, median (IQR)**	10 (2)	10 (2)	10 (2)	10 (2)	0.11
**Awakening stroke** **, *n* (%)**	57 (19)	35	13 (23.6)	9 (15.5)	0.49
**Secondary transfer time** **(median, IQR)**	169 (98)	-	149 (87.5)	188 (79)	0.003
**Stroke etiology (TOAST)**					
Cardioembolic, *n* (%)	163 (54.3)	104 (55.6)	26 (47.3)	33 (56.9)	0.83
Atherothrombotic, *n* (%)	58 (19.3)	37 (19.8)	11 (20)	10 (17.2)	0.84
Other determined etiology, *n* (%)	14 (4.7)	11 (5.9)	1 (1.8)	2 (3.4)	0.48
Undetermined etiology, *n* (%)	57 (19)	35 (18.7)	11 (2)	11 (19)	0.84
**Blood biomarkers**					
Platelet volume **≥** 9.6 fL *n* (%)	100 (33.3)	68 (36.4)	19 (34.5)	13 (22.4)	0.20
NT-proBNP, median (IQR), pg/mL	1154 (2275.3)	1024 (2417)	1024 (2417)	1439 (1881)	0.17

* Diagnosed upon admission or during hospitalization. ** Defined as INR < 1.7. mRS, modified Rankin scale; NIHSS, National Institutes of Health Stroke Scale; IQR, interquartile range; DT, direct transfer; NH, nearby hospital; DH, distant hospital; TOAST, trial of Org 10,172 in acute stroke treatment classification; NT-proBNP, N-terminal pro-brain natriuretic peptide.

**Table 2:** The main features of the procedure, in-hospital complications, and 3-month outcomes.

Variable	Total (N = 300)	DT (N = 187)	NH (N = 55)	DH (N = 58)	*p*
**Procedure**					
IVT, *n* (%)	176 (58.7)	111 (59.4)	28 (50.9)	37 (63.8)	0.43
Onset-to-needle time, median (IQR), min	120 (168)	120 (75)	120 (74)	130 (64)	0.32
Door-to-needle time, median (IQR), min	48 (35)	43 (27)	43 (27)	60 (48)	<0.001
Onset-to-groin time, median (IQR), min	250 (234)	204 (197)	204 (196)	328 (151)	<0.001
Door-to-groin time, median (IQR), min	87 (50)	95 (45)	95 (41)	64 (65)	<0.001
MT duration, median (IQR), min	32 (40)	34 (45)	35 (46)	30 (36)	0.77
Passes in MT ≤ 3, *n* (%)	170 (56.7)	99 (52.9)	37 (67.3)	34 (58.6)	0.05
Stent implantation, *n* (%)	45 (15)	30 (16)	9 (16.4)	6 (10.3)	0.59
TICI 2b-3, *n* (%)	226 (75.3)	144 (77)	37 (67.3)	45 (77.6)	0.82
TICI 0, *n* (%)	14 (4.7)	9 (4.8)	2 (3.6)	3 (5.2)	0.62
**In-hospital complications**					
Hemorrhagic transformation *, *n* (%)	49 (16.3)	23 (12.3)	13 (23.6)	13 (22.4)	0.04
Brain edema with midline deviation, *n* (%)	67 (22.3)	39 (20.9)	14 (25.5)	14 (24.1)	0.63
Craniectomy, *n* (%)	8 (2.7)	4 (2.1)	2 (3.6)	2 (3.4)	0.74
Renal insufficiency, *n* (%)	37 (12.3)	29 (15.5)	5 (9.1)	6 (10.3)	0.39
Renal failure, *n* (%)	40 (12.3)	29 (15.5)	5 (9.1)	6 (10.3)	0.39
Lower respiratory tract infection, *n* (%)	68 (22.7)	40 (21.4)	15 (27.3)	13 (22.4)	0.52
Urinary tract infection, *n* (%)	26 (8.7)	13 (6.9)	7 (12.7)	6 (10.3)	0.3
**Outcomes**					
Early neurological improvement **, *n* (%)	39 (13)	25 (13.6)	11 (20)	3 (5.2)	0.06
Excellent outcome (mRS score = 0–1), *n* (%)	72 (24)	54 (28.9)	10 (18.2)	8 (13.8)	0.07
Favorable outcome (mRS score = 0–2), *n* (%)	106 (35.3)	78 (41.7)	15 (27.3)	13 (22.4)	0.03
Death, *n* (%)	63 (21)	39 (20.9)	10 (18.2)	14 (24.1)	0.71

* Parenchymal hematoma type 1 (PH1) or type 2 (PH2) according to the ECASS classification. ** Improvement in the NIHSS score of 4 or more points after MT. IVT, intravenous thrombolysis; IQR, interquartile range; DT, direct transfer; NH, nearby hospital; DH, distant hospital; MT, mechanical thrombectomy; TICI, thrombolysis in cerebral infarction scale score; NIHSS, National Institutes of Health Stroke Scale; mRS, modified Rankin scale.

**Table 3:** Multivariate analyses of factors associated with functional independence at 3 months (mRS score = 0–2).

Variable	Unadjusted Analysis		Adjusted Analysis ^†^	
	OR (95% CI)	*p*	OR (95% CI)	*p*
**Demographics and comorbidities**				
Age	0.95 (0.93–0.97)	<0.001	0.95 (0.92–0.98)	0.001
Previous mRS score ≤ 1	5.89 (1.73–20.06)	0.005	-	-
Diabetes mellitus	0.64 (0.37–1.103)	0.108	-	-
Atrial fibrillation	0.61 (0.33–1.108)	0.104	-	-
Drug abuse	6.59 (0.72–54.94)	0.09	-	-
**Prior treatments**				
Statins	0.71 (0.46–1.17)	0.18	-	-
Anticoagulants	0.56 (0.26–1.21)	0.14	-	-
Underdosed vitamin K antagonist *	0.23 (0.041–1.28)	0.093	-	-
**Transfer model**				
Direct transfer	Reference		-	-
NH	2.35 (1.17–4.72)	0.016	-	-
DH	1.37 (0.59–3.3)	0.48	0.37 (0.14–0.97)	0.04
**NIHSS**	0.9 (0.86–0.94)	<0.001	0.91 (0.86–0.96)	0.001
**Secondary transfer time**	0.99 (0.99–1.001)	0.19		
**Stroke etiology (TOAST)**				
Other determined etiology	2.17 (0.73–6.45)	0.16	-	-
**Blood biomarkers**				
NT-proBNP	1.00 (1.00–1.00)	0.012	-	-
**Procedure**				
IVT	1.59 (0.97–2.64)	0.068	-	-
Door-to-needle time	1.001 (0.99–1.005)	0.70	-	-
Onset-to-groin time	0.99 (0.99–1.00)	0.13	-	-
Door-to-groin time	1.00 (0.99–1.003)	0.86	-	-
MT duration	0.99 (0.99–1.003)	0.26	-	-
**Passes in MT ≤ 3**	2.87 (1.36–6.06)	0.006	3.8 (1.5–9.6)	0.004
TICI 2b-3	2.8 (1.28–6.19)	0.01	-	-
**In-hospital outcomes and complications**				
Early neurological improvement **	0.105 (0.031–0.35)	<0.001	-	-
Hemorrhagic transformation ***	0.19 (0.077–0.46)	<0.001	-	-
Brain edema with midline deviation	0.087 (0.33–0.23)	<0.001	0.13 (0.038–0.42)	0.001
Craniectomy	0.00 (0.00–0.00)	0.99	-	-
Renal insufficiency	0.53 (0.25–1.16)	0.11	-	-
Lower respiratory tract infection	0.11 (0.047–0.28)	<0.001	0.18 (0.06–0.55)	0.002

* Defined as INR < 1.7. ** Improvement in the NIHSS score of 4 or more points 24 h after MT. *** Parenchymal hematoma type 1 (PH1) or type 2 (PH2) according to the ECASS classification. mRS, modified Rankin scale; NT-proBNP, N-terminal pro-brain natriuretic peptide; NH, nearby hospital; DH, distant hospital; NIHSS, National Institutes of Health Stroke Scale; IVT, intravenous thrombolysis; MT, mechanical thrombectomy; TICI, thrombolysis in cerebral infarction scale score. ^†^ Adjusted by age, previous mRS score ≤ 1, diabetes mellitus, atrial fibrillation, drugs, statins, anticoagulants, underdosed vitamin K antagonist, transfer model, NIHSS, secondary transfer time, stroke etiology, NT proBNP, IVT, door-to-needle time, onset-to-groin time, door-to-groin time, MT duration, number of passes in MT, TICI 2b-3, early neurological improvement, hemorrhagic transformation, brain edema with midline deviation, craniectomy, renal insufficiency, and respiratory infections.

## Data Availability

The data presented in this study are available from the corresponding author upon request. The data are not available to the public due to personal data protection.
